# Psychometric Analyses of the Italian 8-Item, 9-Item, and 12-Item Versions of the Depression, Stress and Anxiety Scale

**DOI:** 10.1177/01632787251380550

**Published:** 2025-09-20

**Authors:** Paolo Soraci, Mark D. Griffiths, Elena Del Fante, Renato Pisanti, Giulia Marafioti, Rocco Servidio, Elisa Chini, Attila Szabo

**Affiliations:** 1 Department of Economics, Psychology, Communication, Education, and Motor Sciences, University “Niccolò Cusano”, Rome, Italy; 2 International Gaming Research Unit, Psychology Department, 6122 Nottingham Trent University, Nottingham, UK; 3 Play Better Associazione, Pistoia, Italy; 4 UniMiB Università degli Studi Milano-Bicocca, Milan, Italy; 5 120349 IRCCS Centro Neurolesi “Bonino-Pulejo”, Messina, Italy; 6 Department of Culture, Education and Society, 18950 University of Calabria, Arcavacata di Rende, Italy; 7 Independent Researcher, Rome, Italy; 8 Faculty of Sport and Health Sciences, 72399 Széchenyi István University, Győr, Hungary

**Keywords:** DASS-12, DASS-9, DASS-8, Italian, psychometric evaluation

## Abstract

The present study aimed to validate the Italian 8-item, 9-item, and 12-item versions of the Depression, Anxiety and Stress Scale-21 (DASS-21), addressing the need for shorter yet psychometrically robust measures. Two studies were conducted with different samples. In Study 1 (*n* = 541), confirmatory factor analysis (CFA) and reliability testing of the short-form versions of the DASS were performed, and their convergent validity with life satisfaction and mental well-being was examined. Study 2 (*n* = 321) extended this validation by reassessing factor structure, reliability, and convergent validity using constructs associated with psychological distress, including positive and negative affect, self-esteem, and perceived stress. Results demonstrated that all short-form versions retained the three-factor structure of the original DASS-21, with overall sufficient fit indices, especially the 9-item model. Reliability metrics confirmed internal consistency (all Cronbach’s alpha and McDonald’s omega ≥0.70). Convergent validity analyses indicated strong correlations between the short-form versions of DASS-21 (min = 0.675, max = 0.956) and associated psychological constructs, aligning with theoretical expectations. The scales captured the relationships between psychological distress, positive and negative affect, perceived stress, mental well-being, self-esteem, and life satisfaction. Findings suggest that the Italian versions of the DASS-8, DASS-9, and DASS-12 provide feasible and reliable alternatives to the DASS-21 for assessing depression, anxiety, and stress, supporting their usefulness in clinical and research contexts, particularly in circumstances in which brevity is essential.

## Introduction

Depression, anxiety, and stress are among the most common mood disorders, with increasing rates worldwide (World Health Organization, 2022). These conditions can negatively impact self-esteem, mental health, and overall life satisfaction (Bandelow & Michaelis, 2015; Ceri & Cicek, 2021; Diener et al., 1985; Keyes, 2006; Lim et al., 2018; Monroe & Cummins, 2015; Sowislo & Orth, 2013). A meta-analysis of 17 studies estimated prevalence rates of 33.7% for depression, 31.9% for anxiety, and 29.6% for stress (Salari et al., 2020). In Italy, Mazza et al. (2020) found very high levels of these symptoms (11%–15%) among 2,766 participants. Although likely influenced by the COVID-19 pandemic, these figures represent the most recent national data available.

These psychological conditions can significantly impair quality of life and daily functioning by reducing positive affect (e.g., enthusiasm, determination) and increasing negative emotions (e.g., distress, nervousness; Wilmer et al., 2021). Depression is associated with elevated suicidal ideation and attempts (Riera-Serra et al., 2024). In Italy, 529 of the 779 suicides reported in 2021 were among individuals with depression (Statista, 2024). Anxiety is associated with poor sleep and eating disorder symptoms (Perez et al., 2023; Zou et al., 2020), while stress is associated with cognitive impairment and social withdrawal (Birmingham & Holt-Lunstad, 2018; Christensen et al., 2023). Given these impacts, accurate assessment of depression, anxiety, and stress is crucial for diagnosis, intervention, and monitoring (Coelho et al., 2020; Monteiro et al., 2022). A widely used instrument for this purpose is the 21-item Depression, Anxiety, and Stress Scale (DASS-21; Lovibond & Lovibond, 1995), a brief version of the original 42-item DASS.

A systematic review of DASS-21 showed it had high-quality psychometric measurement properties (Lee et al., 2019). Its Italian version also has good psychometric properties (Bottesi et al., 2015). However, in response to the need for shorter instruments that save time and reduce the burden on participants (Coelho et al., 2020; Monteiro et al., 2022), the present study aimed to examine the Italian version of the short-form versions of the DASS-21 to establish their psychometric properties with a view to clinical and research application within the Italian population.

### The Tripartite Model of Anxiety and Depression, and DASS

The Depression, Anxiety and Stress Scale (DASS) is based on the tripartite model of affective disorders (Lovibond & Lovibond, 1995), which distinguishes between negative affect (common to both anxiety and depression), physiological hyperarousal (specific to anxiety), and anhedonia (specific to depression). Although these conditions overlap, traditional assessments have struggled to clearly separate them (Antony et al., 1998). To address this, Lovibond and Lovibond (1995) developed the DASS, introducing a third dimension (i.e., stress) to assess symptoms such as tension, irritability, and difficulty relaxing. The DASS has become a widely used instrument for assessing negative emotional states (Szabó, 2010), with the original validation study being cited over 17,000 times by 2025. The DASS provides clinical validity that minimizes the risk of misclassifying these conditions, which can hinder effective treatment. Empirical research across diverse populations, including in Italy, supports its validity and reliability (Bottesi et al., 2015; Ruiz et al., 2017; Wang et al., 2016), enhancing its utility in both research and clinical practice.

The DASS has two main versions: the 42-item version and a shorter 21-item (DASS-21). These instruments evaluate seven aspects of depression (dysphoria, hopelessness, devaluation of life, self-deprecation, lack of interest, anhedonia, and inertia), four aspects of anxiety (autonomic arousal, skeletal muscle effects, situational anxiety, and subjective anxious affect), and five aspects of stress (difficulty relaxing, nervous arousal, being easily upset, irritability, and impatience). The DASS-21, which is preferred in both research and clinical settings, has shown excellent psychometric properties, including high internal consistency (Cronbach’s α > .90 across subscales; Henry & Crawford, 2005) and a stable factor structure (Lovibond & Lovibond, 1995; Osman et al., 2012). It also demonstrates strong convergent validity with related measures such as the Beck Depression Inventory, the State-Trait Anxiety Inventory, and the Perceived Stress Scale (Antony et al., 1998).

Additionally, it has lower correlations with unrelated constructs, supporting its divergent validity (Crawford & Henry, 2004). The scale has been translated and validated in various languages and cultural contexts—including Spanish, Chinese, French, Arabic, Brazilian Portuguese, and Italian—while maintaining strong psychometric performance (Bados et al., 2005; Chen et al., 2023; Moussa et al., 2017; Oei et al., 2013; Vignola & Tucci, 2014). Overall, these features highlight the reliability, validity, and versatility of the DASS-21 across various populations.

### Short-Form Versions of the DASS

Given the established utility of the 42-item and 21-item DASS (Osman et al., 2012), and increasing demand for concise research tools (Rammstedt & Beierlein, 2014), shorter versions of the DASS-21 have been developed. These abbreviated forms reduce research costs—especially when participant compensation is time-based (Monteiro et al., 2023)—and are practical for large-scale studies (Coelho et al., 2020; Eisele et al., 2022). Shorter surveys also enhance participant engagement on unpaid platforms, minimizing fatigue and dropout (Coelho et al., 2020; Eisele et al., 2022). However, maintaining psychometric integrity remains critical (Monteiro et al., 2023).

Short versions, such as DASS-8 (Ali et al., 2021), DASS-9 (Kyriazos et al., 2018; Yusoff, 2013), and DASS-12 (Monteiro et al., 2023), have demonstrated good reliability and both convergent and divergent validity (Kyriazos et al., 2018). However, only the DASS-42 and DASS-21 have been validated in Italian (Bottesi et al., 2015). The lack of validated short forms in Italian limits their use in time-sensitive settings such as large-scale surveys or clinical practice. It also hinders data comparability with international studies and may contribute to higher dropout rates in resource-constrained contexts (Coelho et al., 2020; Eisele et al., 2022).

### The Present Study

To fill this gap (i.e., the absence of Italian versions of the DASS-8, DASS-9, and DASS-12), two studies with different samples were conducted to validate these short-form versions. Each study included both a confirmatory factor analysis (CFA) and an assessment of convergent validity, but using different sets of external variables. This approach allowed for the evaluation of the structural consistency of the short-form versions and for the examination of their associations with a broader range of related psychological constructs, to maximize the robustness and generalizability of the psychometric evidence This two-step strategy aimed to address three key psychometric priorities: (i) *replicability* – to confirm that the factorial structure of the short forms was stable across samples; (ii) *generalizability of convergence –* to demonstrate that the scales maintained consistent associations with different sets of related variables, thereby reducing the likelihood of spurious findings due to sample- or measure-specific effects; and (iii) *alignment with best practices in scale validation –* to follow a sequential validation approach that integrated structural confirmation and validation across multiple external variables, in line with established guidelines in the literature (DeVellis, 2017; Hambleton et al., 2004).

In Study 1, confirmatory factor analysis (CFA) and reliability analyses of the DASS-8, DASS-9, and DASS-12 were conducted, comparing them to the existing 21-item version to ensure psychometric equivalency. Additionally, to ensure that reducing the scale did not diminish the quality of information, correlations between the short-form versions and the DASS-21 with various relevant associated constructs (e.g., satisfaction with life and mental well-being), assessing convergent validity were examined. A similar pattern and strength of correlations were anticipated.

Study 2 examined the DASS-8, DASS-9, and DASS-12 structures, comparing them to the Italian DASS-21. CFA and reliability testing were used to gather additional evidence of factor stability for the new three abbreviated models. Additionally, convergent validity was re-examined by assessing correlations with relevant constructs associated with anxiety, stress, and depression (e.g., positive and negative affective states, mental well-being, self-esteem, perceived stress), to confirm that these short-form scales retained strong associations with related psychological dimensions. Like Study 1, a similar pattern of correlation across all short-form versions of the DASS-21 was expected.

More specifically, it was hypothesized that the shorter versions of the DASS-21 would have (i) sufficient three-factor psychometric structures and adequate reliability (H_1_); (ii) a positive association with negative affect (H_2_); (iii) a positive association with perceived stress (H_3_); (iv) a negative association with mental well-being (H_4_); (v) a negative association with self-esteem (H_5_); (vi) a negative association with positive affect (H_6_); and (vi) a negative association with life satisfaction (H_7_). Together, these studies provide complementary and rigorous evidence supporting the psychometric adequacy of the DASS-8, DASS-9, and DASS-12 in the Italian context.

## Methods (Study 1)

### Sample Size

The sample size required for confirmatory factor analysis (CFA) depends on the number of observed variables in the model. For the DASS-21, which comprises 21 observed variables divided across three factors, guidelines such as those by Kline (2016) and MacCallum et al. (1999) recommend participant-to-variable ratios ranging from 5 to 20 participants per variable. This means that for DASS-21, a minimum of 105 participants (5 per variable), a moderate sample of 210 participants (10 per variable), or an ideal sample size of 420 participants (20 per variable) would be needed to ensure adequate power and reliable results. Similarly, for the shorter versions of the scale, including the DASS-8 (eight items), DASS-9 (nine items), and DASS-12 (12 items), the required sample sizes would follow the same rule of thumb (i.e., 40, 80, or 160 participants for the DASS-8; 45, 90, or 180 participants for the DASS-9; and 60, 120, or 240 participants for DASS-12). Based on participant-to-variable ratios, these thresholds align with evidence that higher ratios provide better stability and generalizability of CFA results, particularly for smaller scales or models with fewer variables (MacCallum et al., 1999).

### Ethics

The present study adhered to the Declaration of Helsinki guidelines involving human participants and received approval from the Ethical Committee of the first author’s university ethics committee. Prior to participation, all participants provided informed consent. Participant identities remained anonymous, and data were securely stored in an encrypted online repository, accessible solely to the research team.

### Participants and Procedure

Participants were recruited through various online forums and social media platforms in Italy (e.g., *Facebook, WhatsApp, Telegram, Instagram*) using a link directing them to a survey hosted on *Google Forms*. The research team distributed this link, inviting individuals to participate voluntarily and anonymously without any incentives. Eligibility criteria included being (i) at least 18 years of age, (ii) an Italian-speaking citizen, and (iii) agreeing to participate by clicking a ‘yes’ button regarding informed consent, prior to being given access to the survey. Over three months (from January 2024 to March 2024), 541 individuals started the online survey, which took an average of 20 to 25 minutes to complete. The average age among participants was 35.36 years (SD = ± 12.14, min = 19, max = 71). Of these, 77.0% were female (*n* = 416), and 23% were male (*n* = 124). Most participants were married or cohabited (37.5%, *n* = 203), followed by being engaged (28.3%, *n* = 153), single (25.7, *n* = 139), or other (9.5, *n* = 51). Regarding educational qualification, more than half of the participants had secondary school education (54.7%, *n* = 296), followed by a bachelor’s degree or higher (43.3%, *n* = 234), and secondary school diploma or lower (2%, *n* = 10).

### Measures

*Depression Anxiety Stress Scale-21* (DASS-21, Henry & Crawford, 2005, Italian version: Bottesi et al., 2015): The DASS-21 was used to assess psychological distress. Participants rated each item on a four-point scale ranging from 0 (*not at all*) to 3 (*very much*), covering three domains: depression (e.g., a lack of excitement), anxiety (e.g., symptoms of an approaching panic attack), and stress (e.g., difficulty in relaxing). Domain scores range from 0 to 21, while the total score, representing overall psychological distress, is calculated by summing the scores of the three domains and ranges from 0 to 63. Higher scores in each domain indicate higher levels of depression, anxiety, and stress. Internal consistency in the present study was very good to excellent, with Cronbach’s alpha values of α = 0.919 for depression, α = 0.892 for anxiety, and α = 0.913 for stress, and McDonald’s omega values of ω = 0.921, ω = 0.897, and ω = 0.914, respectively. For the overall DASS-21, the internal reliability was: α = 0.958 and ω = 0.957.

*Depression Anxiety Stress Scale-8* (DASS-8, (Ali et al., 2021): The participants responded to items on DASS-21, and the responses were then used to calculate scores for the eight-item scale (by using Items 10, 13, and 16 for depression, Items 9, 15, and 20 for anxiety, and Items 8 and 12 for stress (see Appendix A for details) without requiring participants to respond to the DASS-8 items separately. Domain scores range from 0 to 9 for anxiety and depression and from 0 to 6 for stress. In contrast, the total score, representing overall psychological distress, is calculated by summing the scores of the three domains and ranges from 0 to 24.

*Depression Anxiety Stress Scale-9* (DASS-9, Kyriazos et al., 2018; Yusoff, 2013): The participants responded to the DASS-21, and the responses were then used to calculate scores for the nine-item scale (by using Items 5, 10, and 16 for depression, Items 7, 9, and 15 for anxiety, and Items 6, 11, and 14 for stress (see Appendix A for details) without requiring participants to respond to the DASS-9 separately. Domain scores range from 0 to 9, while the total score, representing overall psychological distress, is calculated by summing the scores of the three domains and ranges from 0 to 27.

*Depression Anxiety Stress Scale-12* (DASS-12, Monteiro et al., 2023): The participants responded to the DASS-21, and the responses were then used to calculate scores for the 12-item scale (by using Items 10, 16, 17, and 21 for depression, Items 7, 9, 19, and 20 for anxiety, and Items 1, 8, 11, and 12 for stress (see Appendix A for details) without requiring participants to respond to the DASS-12 separately. Domain scores range from 0 to 12, while the total score, representing overall psychological distress, is calculated by summing the scores of the three domains and ranges from 0 to 36.

*Short Warwick-Edinburgh Mental Well-being Scale* (SWEMWBS, Tennant et al., 2007, Italian version Soraci et al., 2024): The SWEMWBS is a shorter version of the Warwick-Edinburgh Mental Well-Being Scale, consisting of seven positively worded items, and was used to assess mental well-being. Participants rate each item on a seven-point scale ranging from 1 (*none of the time*) to 5 (*all times*). An example of an item is “*I’ve been thinking clearly*.” The total score, representing overall mental well-being, is obtained by summing the scores of all items, with possible scores ranging from 7 to 35. Higher scores indicate greater mental well-being. Internal consistency in the present study was very good, with Cronbach’s α = 0.886 and McDonald’s ω = 0.889.

*Satisfaction With Life Scale* (SWLS, Diener et al., 1985, Italian version di Fabio & Palazzeschi, 2012): The five-item SWLS assessed life satisfaction. Participants rate each item on a seven-point scale, ranging from 1 (*totally disagree*) to 7 (*fully agree*). An example item is “*Most aspects of my life are as I want them to be*.” The total score, obtained by summing the individual item scores, ranges from 7 to 35, with higher scores indicating greater life satisfaction. Internal consistency in the present study was very good with Cronbach’s α = 0.894, and McDonald’s ω = 0.894.

### Data Analysis

The data’s normality was assessed following Muthén and Kaplan’s (1985) guidelines, which recommend that item skewness and kurtosis ideally fall within a ±1 range or at least should not exceed values of ±2 for skewness and ±7 for kurtosis (Kline, 2016, 2023). Subsequent analyses included: (i) descriptive statistics for DASS-8, DASS-9, and DASS-12 (e.g., means, standard deviations) and (ii) evaluation of internal consistency via Cronbach’s alpha, McDonald’s omega, and composite reliability (CR), with values of 0.70 or above considered acceptable (Cheung et al., 2024; McDonald, 1999). The factorial structure and dimensionality of DASS-8, DASS-9, and DASS-12 (see Image S1 to S3 in Supplementary Materials) were evaluated through confirmatory factor analysis (CFA). These were compared to the 21-item version, for which a CFA was also conducted in the present study.

To evaluate model fit, specific indices were used: NNFI (non-normed fit index ≥0.95, with a minimum threshold of 0.90), CFI (comparative fit index ≥0.95, minimum acceptable value 0.90), RMSEA (root mean square error of approximation ≤0.08, maximum acceptable value 0.10), and SRMR (standardized root mean square residual ≤0.06, maximum threshold 0.08) (Hu & Bentler, 1999; Kline, 2016, 2023). Convergent and divergent validity analyses were performed using Pearson correlations (Campbell & Fiske, 1959; Cohen et al., 2003) between the DASS-8, DASS-9, and DASS-12, as well as other psychometric measures (i.e., SWLS and SWEMWBS). Moreover, for adequate discriminant validity, the HTMT (heterotrait-monotrait) ratio between two constructs should not exceed 0.85, although other authors indicate that values up to 0.90 may be acceptable (Henseler et al., 2014).

Missing data were below the recommended thresholds (<5%) and were missing completely at random, as indicated by Little (1988). The pairwise technique was used to handle the missing data (Kang, 2013). Data analyses used the R Core Team package R Core Team (2021) with lavaan (version 0.6-19, Rosseel, 2012) for confirmatory factor analysis, and JASP version 0.19 (JASP Team, 2024) for descriptive and correlational analyses.

### Convergent and Divergent Validity

To provide robust, theory-driven evidence of convergent and divergent validity for the DASS-8, DASS-9, and DASS-12, five psychometric instruments were selected that collectively span positive well-being, negative distress, and core intrapsychic resources. Each of the selected instruments has established psychometric properties among Italian samples (α values >0.80) and comprise constructs with clear conceptual links to depression, anxiety, or stress. More specifically, the Satisfaction With Life Scale (SWLS) and Short Warwick–Edinburgh Mental Well-Being Scale (SWEMWBS) assess global well-being, which were predicted to correlate negatively with total DASS scores. The Rosenberg Self-Esteem Scale (RSES) assesses self-worth, and was expected to correlate negatively with depression and anxiety symptoms. The Positive and Negative Affect Schedule (PANAS) assesses both positive-affect deficits and negative-affect elevations, and was expected to negatively and positively correlate with total DASS scores, respectively. The Perceived Stress Scale-10 (PSS-10) assesses perceived stress, and was expected to positively correlate with scores on the DASS stress dimension. By using different instruments in Study 1 (SWLS, SWEMWBS) and Study 2 (RSES, PANAS, SWEMWBS, PSS-10), the overall investigation ensured (i) *breadth* – those convergent correlations held across multiple facets of mental health, and (ii) *replicability* – that these associations were not idiosyncratic to any single criterion scale. This approach follows best practices in scale validation by combining theoretical relevance, psychometric soundness, and cultural appropriateness.

## Results (Study 1)

### Descriptive Analysis

The descriptive statistics indicated that the mean score on the (i) SWLS was 23.15 out of 35 (SD = ± 6.72), (ii) DASS-21 was 24.79 out of 63 (SD = ± 14.88), (iii) DASS-8 was 9.32 out of 24 (SD = ± 6.40), (iv) DASS-9 was 10.39 out of 27 (SD = ± 6.81), (v) DASS-12 was 15.24 out of 36 (SD = ± 8.71), and (vi) SWEMWBS was 25.32 out of 35 (SD = ± 5.30).

### Confirmatory Factor Analysis

Regarding the distribution of the DASS-21 items, all items fell within the optimal values considered to be normally distributed, both for skewness (min = 0.201, max = 1.032, in absolute value) and kurtosis (min = 0.237, max = 1.133, in absolute value) (for details see Table S1 in the Supplementary Materials).

For the DASS-8, the CFA indicated a good fit to the data: χ^2^ = 68.712 (df = 15, *n* = 541; with χ^2^/df = 4.60); *p* < 0.001, CFI = 0.978, NNFI = 0.959, RMSEA = 0.083, 90% CI (0.064, 0.104), *p* < 0.05, SRMR = 0.026, GFI = 0.967. All factor loadings exhibited high and statistically significant values for all items (min = 0.70, max = 0.92; i.e., λij ≥0.50). The AVE for depression was 0.689, anxiety was 630, and stress was 0.637. The ECVI was 0.214.

For the DASS-9, the CFA indicated a good fit to the data: χ^2^ = 67.838 (df = 23, n = 541; with χ^2^/df = 2.99); *p* < 0.001, CFI = 0.981, NNFI = 0.972, RMSEA = 0.062, 90% CI (0.045, 0.079), *p* > 0.05, SRMR = 0.027, GFI = 0.970. All factor loadings exhibited high and statistically significant values for all items (min = 0.66, max = 0.88; i.e., λij ≥0.50). The AVE for depression was 0.802, anxiety was 0.597, and stress was 0.548). The ECVI was 0.214.

For the DASS-12, the CFA indicated a good fit to the data: χ^2^ = 194.48 (df = 51, n = 541; with χ^2^/df = 3.81); *p* < 0.001, CFI = 0.964, NNFI = 0.953, RMSEA = 0.074, 90% CI (0.063, 0.085), *p* < 0.05, SRMR = 0.035, GFI = 0.943. All factor loadings exhibited high and statistically significant values for all items (min = 0.67, max = 0.89; i.e., λij ≥0.50). The AVE for depression was 0.688, anxiety was 0.562, and stress was 0.650. The ECVI was 0.481.

For the DASS-21, the CFA indicated a good fit to the data: χ^2^ = 783.422 (n = 541, df = 186, *p* < 0.001; χ^2^/df = 4.21); CFI = 0.925; NNFI = 0.915; RMSEA = 0.078 (90% CI = 0.072, 0.083, *p* < 0.001); SRMR = 0.047; GFI = 0.918. All factor loadings exhibited high and statistically significant values for all items (min = 0.70, max = 0.86; i.e., λij ≥0.50). The AVE for depression was 0.626, anxiety was 0.564, and stress was 0.605. The ECVI was 1.724. For further details, see Table 1 and Tables S2 to S5 in the Supplementary Materials. The results of the HTMT ratio analyses showed that no value in the short versions of the DASS (i.e., DASS-8, DASS-9, and DASS-12) exceeded 0.85 (for details, see Tables S12 to S15 in the Supplementary Materials).

**Table 1:** Study 1. CFA Model Comparison of DASS-8, DASS-9, DASS-12, and DASS-21

Metric	DASS-8	DASS-9	DASS-12	DASS-21
Chi-square (χ^2^)	68.712	67.838	194.484	783.422
*df* (degrees of freedom)	15	23	51	186
Root mean square error of approximation	0.083	0.062	0.074	0.078
Root mean square error of approximation 90% CI	0.064 - 0.104	0.045 - 0.079	0.063 - 0.085	0.072 - 0.083
Bentler-bonett non-normed fit index	0.959	0.972	0.953	0.915
Standardized root mean square residual	0.026	0.027	0.035	0.047
Expected cross validation index	0.214	0.214	0.481	1.724
Goodness of fit index	0.967	0.970	0.943	0.918

### Correlation Analyses

The correlations between stress, anxiety, and depression assessed with the three short-form versions of the DASS showed significant relationships between stress, anxiety, and depression, as well as their negative associations with mental well-being and life satisfaction (Table 2). More specifically, the intercorrelations yielded the following results. In the 8-item version, stress was significantly correlated with anxiety (*r* = 0.671, *p* < 0.001) and depression (*r* = 0.692, *p* < 0.001), and anxiety was significantly correlated with depression (*r* = 0.680, *p* < 0.001). In the 9-item version, the intercorrelations were slightly higher for stress than for anxiety-depression: stress ↔ anxiety (*r* = 0.718, *p* < 0.001), stress ↔ depression (*r* = 0.711, *p* < 0.001), and anxiety ↔ depression (*r* = 0.645, *p* < 0.001). Finally, in the 12-item version, the intercorrelations were high between all pairs: stress ↔ anxiety (*r* = 0.810, *p* < 0.001), stress ↔ depression (*r* = 0.816, *p* < 0.001), and anxiety↔ depression (*r* = 0.823, *p* < 0.001).

**Table 2:** Correlations Between DASS Versions (DASS-21, DASS-8, DASS-9, and DASS-12), Life Satisfaction and Mental Well-Being

Variable	1	2	3	4	5	6	7	8	9	10	11	12	13	14
1. Stress (DASS-21)	—													
2. Anxiety (DASS-21)	0.771***	—												
3. Depression (DASS-21)	0.775***	0.731***	—											
4. Stress (DASS-8)	0.925***	0.694***	0.714***	—										
5. Anxiety (DASS-8)	0.744***	0.937***	0.719***	0.671***	—									
6. Depression (DASS-8)	0.751***	0.679***	0.956***	0.692***	0.680***	—								
7. Stress (DASS-9)	0.954***	0.739***	0.753***	0.822***	0.710***	0.733***	—							
8. Anxiety (DASS-9)	0.735***	0.936***	0.692***	0.652***	0.925***	0.648***	0.718***	—						
9. Depression (DASS-9)	0.727***	0.675***	0.950***	0.666***	0.677***	0.938***	0.711***	0.645***	—					
10. Stress (DASS-12)	0.865***	0.848***	0.783***	0.749***	0.773***	0.713***	0.847***	0.838***	0.709***	—				
11. Anxiety (DASS-12)	0.835***	0.866***	0.842***	0.767***	0.770***	0.816***	0.808***	0.759***	0.795***	0.810***	—			
12.Depression (DASS-12)	0.911***	0.809***	0.816***	0.916***	0.814***	0.756***	0.842***	0.796***	0.808***	0.816***	0.823***	—		
13. SWL	−0.432***	−0.386***	−0.550***	−0.381***	−0.363***	−0.539***	−0.420***	−0.383***	−0.505***	−0.439***	−0.482***	−0.420***	—	
14. MWB	−0.486***	−0.419***	−0.577***	−0.425***	−0.405***	−0.563***	−0.478***	−0.410***	−0.581***	−0.457***	−0.506***	−0.503***	0.627***	—

*Note.* ***p < 0.001. SWL = Satisfaction with life. MBW = Mental well-being.

All versions of the DASS showed significant negative associations with life satisfaction (on the SWLS) and mental well-being (on the SWEMWBS). In all four versions of the DASS (DASS-21, DASS-12, DASS-9, and DASS-8), stress, anxiety, and stress negatively correlated with life satisfaction and mental well-being (Table 2).

## Methods (Study 2)

### Participants and Procedure

The procedure and eligibility criteria were the same as Study 1. The data collection took place over a four-month period (from November 2024 to February 2025). A total of 323 individuals started the online survey, which took an average of 15 to 25 minutes to finish. In terms of gender distribution, 75.0% were female (*n* = 242), and 25.0% were male (*n* = 80). The average age among participants was 34.38 years (SD = ± 12.83, min = 18, max = 80). Regarding educational background, 52.93% had a university degree or higher (*n* = 171), 46.13% had completed high school (*n* = 149), and 3.0% had a secondary school diploma or lower (*n* = 3). Moreover, 65.01% were single (*n* = 210), 26.31% were married/cohabiting (*n* = 85), and the remaining 8.64% were in another type of relationship (*n* = 28). See Table S11 in the Supplementary Materials for a summary comparison between Study 1 and Study 2 participants.

### Measures

In addition to completing the four versions of the DASS (see ‘Measures’ in Study 1), the following four psychometric scales were included.

*Rosenberg Self-Esteem Scale* (RSES; Rosenberg, 1965; Italian version: Prezza et al., 1997): The 10-item RSES was used to assess self-esteem. The scale includes items such as “*In general, I am satisfied with myself*” with participants indicating their agreement on a four-point Likert scale ranging from 0 (*strongly disagree*) to 3 (*strongly agree*), resulting in total scores from 0 to 30. Higher scores indicate greater self-esteem. Internal consistency in the present study was very good with Cronbach’s α = 0.91, and McDonald’s ω = 0.889.

*Short Warwick-Edinburgh Mental Well-Being Scale* (SWEMWBS; Tennant et al., 2007 – 14 items; Italian version; Soraci et al., 2024): The seven-item Short WEMWBS was used to assess mental well-being. Participants rated items such as “*I’ve been feeling optimistic about the future*” on a five-point scale, ranging from 1 (*never*) to 5 (*always*), resulting in total scores from 7 and 35. Higher scores indicate greater mental well-being. Internal consistency in the present study was very good with Cronbach’s α = 0.867 and McDonald’s ω = 0.867.

*Positive and Negative Affect Schedule* (PANAS, Watson et al., 1988, Italian version (Terracciano et al., 2003): The PANAS was used to assess positive and negative affective states. The scale assesses two independent dimensions: positive affect (PA) and negative affect (NA). It includes 20 adjectives, divided equally between positive and negative affect subscales. The PA subscale represents a person’s level of enthusiasm, energy, and determination, while the NA subscale represents unpleasant emotional states such as anger, guilt and fear. Participants are asked to indicate how much they generally feel in line with each adjective using a five-point Likert scale from 1 (*not at all*) to 5 (*very much*). Some examples of adjectives are “*concerned*,” “*enthusiastic*,” “*determined*,” “*distressed*,” “*hostile*,” and “*nervous*.” Total scores range from 5 to 50 for each subscale. Higher scores on the scales reflect either a more positive or more negative overall affective state. Internal consistency in the present study was very good for both positive affect (Cronbach’s α = 0.925 and McDonald’s ω = 0.928) and negative affect (Cronbach’s α = 0.898 and McDonald’s ω = 0.905).

*Perceived Stress Scale*-*10* (PSS-10, Cohen et al., 1983, Italian version Mondo et al., 2021): The 10-item Perceived Stress Scale was used to assess perceived stress. The scale assesses the extent to which individuals perceive situations in their lives as stressful, considering factors such as lack of control and unpredictability of events. Items are rated on a five-point Likert scale, from 0 (*never*) to 4 (*very often*). Examples of items include questions such as “*How often did you feel unable to control the important things in your life?*” and “*How often did you feel that difficulties were piling up to the point that you could not handle them?*” The total scores range from 0 to 40. Higher scores indicate greater perceived stress. Internal consistency in the present study was good with Cronbach’s α = 0.701, and McDonald’s ω = 0.706.

### Data Analysis

In Study 2, the same types of data analysis were carried out as in Study 1, except for convergent and divergent validity analysis, which was performed using Pearson’s correlations (Campbell & Fiske, 1959; Cohen et al., 2003) between the DASS-8, DASS-9, and DASS-12 tests and other different psychometric measures (i.e., RSES, PSS, PANAS, SMWB).

## Results (Study 2)

The descriptive statistics indicated that the mean score on the (i) PSS was 29.76 out of 40.00 (SD = ± 5.84), (ii) PANAS positive affect was 34.17 out 50.00 (SD = ± 8.27), (iii) PANAS negative affect was 24.42 out 50 (SD = ± 9.83), RSES was 16.11 out 30.00 (SD = ± 2.89), (iv) SWMBS was 24.40 out 35.00 (SD = ± 5.81), (v) DASS-21 was 49.56 out of 63 (SD = ± 21.83), (vi) DASS-8 was 19.31 out of 24 (SD = ± 9.16), (vii) DASS-9 was 21.27 out of 27 (SD = ± 9.64), and (viii) DASS-12 was 28.56 out of 36 (SD = ± 12.86).

### Confirmatory Factor Analysis

With regards to the distribution of the DASS-21 items, all items were within the optimal values to be considered as normally distributed, both for skewness (min = 0.234, max = 1.153, in absolute value) and kurtosis (min = 0.327, max = 1.357, in absolute value), for details see Table S7 in the Supplementary Materials. For the DASS-8, the CFA indicated a good fit to the data (except for RMSEA slightly above the limit): χ^2^ = 102.46 (df = 17, *n* = 323; with χ^2^/df = 6.02); *p* < 0.001, CFI = 0.954, NNFI = 0.923, RMSEA = 0.125, 90% CI (0.99, 0.149), *p* < 0.05, SRMR = 0.031, GFI = 0.968. All factor loadings exhibited high and statistically significant values for all items (min = 0.74, max = 0.92; i.e., λij ≥0.50). The AVE for depression was 0.700, anxiety was 645, and stress was 0.718. The ECVI = 0.487.

For the DASS-9, the CFA indicated a good fit to the data: χ^2^ = 86.581 (df = 24, *n* = 323; with χ^2^/df = 3.62); *p* < 0.001, CFI = 0.964, NNFI = 0.949, RMSEA = 0.090, 90% CI (0.070, 0.111), *p* > 0.05, SRMR = 0.030, GFI = 0.973. All factor loadings exhibited high and statistically significant values for all items (min = 0.62, max = 0.84; i.e., λij ≥0.50). The AVE for depression was 0.580, anxiety was 0.587, and stress was 0.668). The ECVI 0.457.

For the DASS-12, the CFA indicated a good fit to the data: χ^2^ = 166.705 (df = 51, *n* = 323; with χ^2^/df = 3.27); *p* < 0.001, CFI = 0.959, NNFI = 0.947, RMSEA = 0.084, 90% CI (0.070, 0.098), *p* < 0.05, SRMR = 0.035, GFI = 0.958. All factor loadings exhibited high and statistically significant values for all items (min = 0.67, max = 0.89; i.e., λij ≥0.50). The AVE for depression was 0.684, anxiety was 0.5742, and stress was 0.729. The ECVI = 0.762.

For the DASS-21, the CFA indicated a fit to the data slightly below the acceptable limits: χ^2^ = 779.466 (df = 186, *n* = 323; χ^2^/df = 4.19), *p* < 0.001; CFI = 0.895; NNFI = 0.882; RMSEA = 0.100 (90% CI = 0.093, 0.107, *p* < 0.001); SRMR = 0.047; GFI = 0.858. All factor loadings exhibited high and statistically significant values for all items (min = 0.70, max = 0.86; i.e., λij ≥0.50). The AVE for depression was 0.628, anxiety was 0.563, and stress was 0.701. The ECVI = 2.839. For further details, see Table 3 and Tables S6 to S10 in the Supplementary Materials. The results of the HTMT ratio analyses showed that no value in the short versions of the DASS (i.e., DASS-8, DASS-9, and DASS-12) exceeded 0.85 (for details, see Tables S16 to S18 in the Supplementary Materials).

**Table 3:** Study 2. CFA Model Comparison of DASS 8, DASS-9, DASS-12 and DASS-21

Metric	DASS-8	DASS-9	DASS-12	DASS-21
Chi-square (*χ*^2^)	102.46	86.581	166.705	779.466
df (degrees of freedom)	17	24	51	186
Root mean square error of approximation	0.125	0.090	0.084	0.100
Root mean square error of approximation 90% CI	(0.099, 0.149)	(0.070, 0.111)	(0.070, 0.098)	(0.093, 0.107)
Bentler-bonett non-normed fit index	0.923	0.949	0.947	0.882
Standardized root mean square residual	0.031	0.030	0.035	0.047
Expected cross validation index	0.487	0.457	0.762	2.839
Goodness of fit index	0.968	0.973	0.958	0.858

### Correlations

The correlations between stress, anxiety, and depression assessed with the three short versions of the DASS showed significant relationships between the three variables, as well as positive associations with mental well-being, positive affect and self-esteem, and their negative associations with perceived stress and negative affect (see Tables 4 and 5 for further details).

**Table 4:** Correlations Between the DASS-8, DASS-9, DASS-12, and DASS-21, Perceived Stress, and Self-Esteem

Variable	1	2	3	4	5	6	7	8	9	10	11	12	13	14
1.Depression-8	—													
2. Anxiety-8	0.781***	—												
3. Stress-8	0.736***	0.731***	—											
4. Depression-9	0.942***	0.740***	0.712***	—										
5. Anxiety-9	0.716***	0.934***	0.728***	0.695***	—									
6. Stress-9	0.797***	0.791***	0.874***	0.778***	0.767***	—								
7. Depression-12	0.950***	0.754***	0.657***	0.920***	0.691***	0.732***	—							
8. Anxiety-12	0.760***	0.926***	0.759***	0.740***	0.919***	0.798***	0.741***	—						
9. Stress-12	0.770***	0.754***	0.969***	0.743***	0.751***	0.914***	0.693***	0.774***	—					
10. Stress-21	0.806***	0.800***	0.950***	0.783***	0.780***	0.968***	0.740***	0.816***	0.976***	—				
11. Anxiety-21	0.774***	0.936***	0.767***	0.762***	0.943***	0.809***	0.752***	0.967***	0.793***	0.825***	—			
12.Depression-21	0.965***	0.777***	0.744***	0.962***	0.733***	0.812***	0.967***	0.780***	0.782***	0.822***	0.795***	—		
13. Perceived stress	0.514***	0.509***	0.564***	0.523***	0.490***	0.570***	0.500***	0.519***	0.580***	0.601***	0.519***	0.533***	—	
14. Self-esteem	−0.261***	−0.247***	−0.253***	−0.235***	−0.233***	−0.257***	−0.244***	−0.250***	−0.262***	−0.264***	−0.249***	−0.258***	−0.226***	—

*Note.* ***p < 0.001.

**Table 5:** Correlations Between DASS-8, DASS-9, DASS-12, DASS-21, Positive and Negative Affect and Mental Well-Being

Variable	1	2	3	4	5	6	7	8	9	10	11	12	13	14	15
1.Depression-8	—														
2. Anxiety-8	0.781***	—													
3. Stress-8	0.736***	0.731***	—												
4.Depression-9	0.942***	0.740***	0.712***	—											
5. Anxiety-9	0.716***	0.934***	0.728***	0.695***	—										
6. Stress-9	0.797***	0.791***	0.874***	0.778***	0.767***	—									
7. Depression-12	0.950***	0.754***	0.657***	0.920***	0.691***	0.732***	—								
8. Anxiety-12	0.760***	0.926***	0.759***	0.740***	0.919***	0.798***	0.741***	—							
9. Stress-12	0.770***	0.754***	0.969***	0.743***	0.751***	0.914***	0.693***	0.774***	—						
10.Stress-21	0.806***	0.800***	0.950***	0.783***	0.780***	0.968***	0.740***	0.816***	0.976***	—					
11.Anxiety-21	0.774***	0.936***	0.767***	0.762***	0.943***	0.809***	0.752***	0.967***	0.793***	0.825***	—				
12.Depression-21	0.965***	0.777***	0.744***	0.962***	0.733***	0.812***	0.967***	0.780***	0.782***	0.822***	0.795***	—			
13.Positive affect	−0.440***	−0.305***	−0.263***	−0.412***	−0.270***	−0.283***	−0.422***	−0.292***	−0.302***	−0.305***	−0.287***	−0.445***	—		
14.Negative affect	0.675***	0.679***	0.630***	0.670***	0.644***	0.666***	0.667***	0.673***	0.650***	0.689***	0.665***	0.695***	−0.339***	—	
15. Mental well-being	−0.652***	−0.549***	−0.529***	−0.634***	−0.498***	−0.550***	−0.637***	−0.542***	−0.562***	−0.580***	−0.536***	−0.669***	0.672***	−0.658***	—

*Note.* ***p < 0.001.

### Relationships Between Stress, Anxiety and Depression

In all versions of the DASS (i.e., DASS-21, DASS-12, DASS-9, and DASS-8), stress and anxiety showed a significant positive correlation, as did stress and depression, and anxiety and depression (see Tables 4 and 5). More specifically, the intercorrelations yielded the following results. In the 8-item version, stress was significantly correlated with anxiety (*r* = 0.731, *p* < 0.001) and depression (*r* = 0.736, *p* < 0.001), and anxiety was significantly correlated and depression (*r* = 0.781, *p* < 0.001). In the 9-item version, the intercorrelations were similar: stress ↔ anxiety (*r* = 0.767, *p* < 0.001), stress ↔ depression (*r* = 0.778, *p* < 0.001), and anxiety ↔ depression (*r* = 0.695, *p* < 0.001). Finally, the 12-item version also showed similar intercorrelations: stress ↔ anxiety (*r* = 0.774, *p* < 0.001), stress ↔ depression (*r* = 0.693, *p* < 0.001), and anxiety ↔ depression (*r* = 0.741, *p* < 0.001).

### Relationships With Perceived Stress

All versions of the DASS showed significant positive associations with perceived stress (i.e., scores on the PSS). For the DASS-21, stress, anxiety, and depression were all positively correlated with perceived stress. Similar patterns were observed for the DASS-12, DASS-9, and DASS-8 where these variables were strongly positively correlated with perceived stress (see Table 4 for further details).

### Relationships With Positive and Negative Affect

All versions of the DASS showed significant positive associations with negative affect and significant negative associations with positive affect (i.e., scores on the PANAS). For the DASS-21, stress, anxiety, and depression correlated positively with negative affect and negatively with positive affect (PANAS scale). Similar patterns were observed for the DASS-12, DASS-9, and DASS-8 (see Table 5 for further details).

### Associations With Self-Esteem and Mental Well-Being

All versions of the DASS showed significant negative associations with mental well-being (i.e., scores on the SMWBS) and self-esteem (i.e., scores on the RSES). For the DASS-21, stress, anxiety, and depression were negatively correlated with self-esteem and with mental well-being. Similar patterns are observed in DASS-12, DASS-9, and DASS-8. The four versions of the DASS showed consistency in their results, highlighting significant positive correlations between stress, anxiety and depression, negative affect, and perceived stress as well as their negative relationships with mental well-being and self-esteem. These results confirm the validity of the different versions of the instrument in assessing psychological constructs (see Tables 4 and 5 for further details).

## Discussion

Across two psychometric evaluations, the present study examined the psychometric properties of three shortened versions of the DASS-21 (DASS-8, DASS-9, and DASS-12) among Italian adults. The results of both studies demonstrated good psychometric properties, which are discussed separately.

### Study 1

The comparison of the new short versions of the DASS-21, focusing on data fit and parsimony, indicated that the DASS-9 was the best model examined. This result aligns with previous literature emphasizing the balance between model complexity and fit (Hu & Bentler, 1999). Both the DASS-8 and DASS-9 presented the lowest ECVI, indicating a higher likelihood of replicability among new samples (Kline, 2016; Marsh et al., 2004). In contrast, both DASS-12 and DASS-21’s ECVI suggested lower replicability.

Evaluating the overall model fit, the DASS-9 showed the highest NNFI compared to the DASS-8, DASS-12, and DASS-21, further positioning the DASS-9 as the best-fitting model (Browne & Cudeck, 1992). DASS-9 also demonstrated a lower RMSEA, outperforming DASS-8, DASS-12, and DASS-21, and underscoring its strong fit regarding approximation error (Steiger, 1990). Similarly, the DASS-9’s GFI was slightly higher than that of the DASS-8, with both exceeding the DASS-12’s and DASS-21’s GFI, reinforcing the conclusion favoring the DASS-9 for its balance of simplicity and fit (Jöreskog & Sörbom, 1993).

In addition, the SRMR highlighted the superior fit of the DASS-8 and DASS-9, which achieved the lowest values, outperforming both the DASS-12 and DASS-21. This further emphasizes the DASS-9’s robustness in terms of overall model fit, followed by the 12-item version of the DASS. Internal consistency on all the versions of the DASS (8, 9, and 12 items) were good. These results support H_1_.

From a theoretical perspective, the DASS-9 is preferable due to its item balance, with three items each for anxiety, stress, and depression, closely mirroring the 21- and 42-item versions of the DASS, which include seven and 14 items per construct, respectively (Lovibond & Lovibond, 1995). While DASS-8 has been criticized for including only two items for stress, potentially limiting its ability to assess this construct fully (Marsh et al., 2004), research supports the use of three items per factor in psychological assessments, further validating the DASS-9 as a theoretical sound model (Kline, 2016). Although DASS-21 maintains greater item coverage across constructs, its increased complexity and weaker fit indices make it less suitable for practical applications, favoring the DASS-9 as an efficient yet comprehensive assessment instrument. In sum, although all the short versions of DASS-21 have overall sufficient fit, the one that balances parsimony and fits indices in the most optimal way is the DASS-9.

Moreover, the three short versions of DASS-21 demonstrated adequate convergent and divergent validity, therefore confirming their ability to consistently assess the dimensions of stress, anxiety and depression, as evidenced in DASS-21. The correlations between these variables (i.e., anxiety, stress and depression) in the short versions follow a similar pattern to that of DASS-21, with significant relationships of comparable magnitude. The average variance extracted (AVE) analysis yielded satisfactory values for each dimension in the short versions, exceeding the recommended threshold of 0.50 (50%). These findings suggest that the short versions adequately capture the variance of the assessed constructs, consistent with the findings of previous studies (e.g., Diener et al., 1985; Henry & Crawford, 2005; Lovibond & Lovibond, 1995; Osman et al., 2012). Moreover, HTMT values were below 0.85 for all short versions of the DASS indicating sufficient discriminant validity.

The associations between dimensions of psychological distress and well-being indicators, such as life satisfaction and mental well-being (which supported H_4_ and H_7_), showed a significant negative pattern in all short versions of the DASS-21. These findings are consistent with previous literature that has extensively documented how high levels of stress, anxiety, and depression are associated with lower perceptions of subjective well-being and lower life satisfaction (e.g., Diener et al., 1985; Henry & Crawford, 2005; Lovibond & Lovibond, 1995; Osman et al., 2012).

In particular, the results suggest that each dimension of psychological distress affects an individual’s well-being in different but complementary ways. For example, depression showed the strongest negative associations with life satisfaction and mental well-being, suggesting that depressed mood and feelings of personal ineffectiveness have a marked effect on perceived quality of life (e.g., Lathabhavan & Sudevan, 2023). On the other hand, stress correlated more closely with feelings of overload and difficulty coping with environmental demands, negatively affecting both satisfaction and mental well-being (e.g., Lathabhavan & Sudevan, 2023). Finally, anxiety appeared to be related to levels of constant worry and hypervigilance, which can interfere with perceived well-being (e.g., Lathabhavan & Sudevan, 2023).

These associations reflect an important theoretical confirmation: indicators of psychological distress and well-being not only influence each other, but constitute complementary dimensions of a mental health continuum, as highlighted in theoretical dual continua models of mental health (Henry & Crawford, 2005; Keyes, 2002). The ability to assess these dimensions with short, validated instruments represents added value for both research and clinical practice. The observed correlation values are consistent with those reported in previous studies (e.g., Monteiro et al., 2023) on the 21-item version and the three short versions of DASS-21, reinforcing the convergent and discriminant validity of the results. Importantly, the short versions DASS-21 do not appear to result in a loss of information quality, as evidenced by the strength and consistency of these correlations. These findings are consistent with previous research supporting the reliability and utility of the DASS in both long and short formats and reinforce its robustness across different model structures (e.g., Monteiro et al., 2023).

### Study 2

Similar to the findings in Study 1, the model comparison of the short versions of DASS-21 indicated that, overall, DASS-9 was the best model. More specifically, the results indicated that DASS-8 and DASS-9 presented the lowest ECVI (Kline, 2016). In contrast, DASS-12 and DASS-21 had a lower replicability, confirming what was found in the first study. Evaluating overall model fit, the DASS-9 showed the highest NNFI compared to the other three versions of the DASS, further positioning the DASS-9 as the best-fitting model (Browne & Cudeck, 1992). The DASS-12 demonstrated a marginally lower RMSEA than the DASS-9, yet both of these measures exhibited superior performance in comparison to the DASS-8 and DASS-21, thereby emphasizing their robust alignment with regard to approximation error (Steiger, 1990). Similarly, DASS-9’s GFI was slightly higher than that of DASS-8, with both exceeding the GFI of both the DASS-12 and DASS-21.

DASS-9 achieved the lowest SRMR, closely followed by DASS-8. The DASS-12 demonstrated a slightly higher SRMR, while the DASS-21 had the highest value, indicating a relatively weaker fit. In sum, although all short versions of the DASS showed sufficient overall fit (although the RMSEA of DASS-8 and DASS-21 were slightly high), the DASS-9 model stood out due to a better balance between parsimony and quality of fit indices, similar to Study 1. Internal consistencies of all the short versions of the DASS-21 were good (which supports H_1_).

Similar to the results of Study 1, the short versions of DASS-21 also showed good convergent and divergent validity in Study 2, confirming their ability to effectively represent the dimensions of stress, anxiety and depression, in line with DASS-21. The correlations between anxiety, stress, and depression in the short versions followed a trend consistent with that observed in DASS-21, being significant and of similar strength. The average variance extracted (AVE) analysis showed adequate values for each dimension in the short versions, exceeding the recommended threshold of 0.50 (50%). These results indicated that the short versions capture a significant proportion of the variance of the assessed constructs, confirming findings from previous research on the longer (DASS-21) version (e.g., Bottesi et al., 2015; Lovibond & Lovibond, 1995; Osman et al., 2012). Similar to Study 1, HTMT values were below 0.85 for all short versions of the DASS indicating sufficient discriminant validity.

Across all short versions of DASS-21, stress, anxiety and depression showed significant positive associations with perceived stress (supporting H_3_) and confirming that higher levels of psychological distress are associated with higher perceived stress in daily life, consistent with previous studies (Bottesi et al., 2015; Lovibond & Lovibond, 1995; Osman et al., 2012). The associations between the DASS-21 dimensions (anxiety, stress, and depression) and affect (as assessed using the PANAS), showed a clear pattern in line with theoretical expectations. More specifically, all short versions of the DASS showed significant positive correlations with negative affect, indicating that higher levels of depression, anxiety, and stress were associated with more negative emotions such as fear, irritation, and sadness.

In contrast, significant negative correlations were observed with positive affect, suggesting that an increase in the psychopathological dimensions of the DASS is associated with a decrease in the frequency of positive emotional experiences such as enthusiasm, gratitude, and joy, in line with previous studies (Crawford & Henry, 2004; Henry & Crawford, 2005; Osman et al., 2012). These results supported H_2_ and H_6_ and provided further empirical evidence for the convergent validity of the short versions of the DASS-21 regarding different emotional constructs. Moreover, these associations confirmed the sensitivity of the short versions in detecting the relationship between psychological distress and emotional patterns, consistent with what has been reported in previous studies (Crawford & Henry, 2004; Gaudreau et al., 2006; Henry & Crawford, 2005). The robustness of these results, also observed for the short versions, supports the possibility of using the shortened DASS-21 versions as efficient instruments for assessing emotional distress and its affective correlates in clinical and research contexts.

The significant negative correlation between the dimensions of stress, anxiety, and depression assessed by all short versions of DASS-21 and mental well-being is particularly pronounced, especially for depression, underlining the profound impact of psychological distress on perceptions of overall well-being. This finding confirms the importance of depression as a determinant of lower mental well-being because this dimension is associated not only with an increase in emotional distress but also with an impairment in the ability to experience positive and satisfying mental states. These findings are also in line with a well-established body of research showing that depression, more than other dimensions of psychological distress, is closely associated with reduced quality of life and perceived well-being (e.g., Yeshaw & Mossie, 2017).

Similarly, the negative associations between the DASS dimensions on the three short versions of the DASS-21 and self-esteem were also significant (supporting H_5_), although lower than for mental well-being, suggesting that psychological distress may undermine positive self-evaluations. This relationship appears to be consistent with previous studies (e.g., Nguyen et al., 2019), which have shown that high levels of stress, anxiety, and depression tend to reduce confidence in an individual’s abilities and promote feelings of inadequacy and personal insecurity (e.g., Gu et al., 2024). This relationship may be particularly critical in clinical settings, where compromised self-esteem may further exacerbate symptoms of psychological distress, creating a vicious cycle that is difficult to break (e.g., Nguyen et al., 2019).

The present study’s findings confirm that the three short versions of DASS-21 consistently assessed the psychological constructs of stress, anxiety, and depression and their relationships with related variables. The correlations between perceived stress, negative and positive affect, self-esteem, and psychological well-being showed a robust pattern consistent with previous studies (Keyes, 2002), emphasizing the importance of psychological distress dimensions in shaping perceptions of well-being. In particular, the short versions of the DASS-21 represent a good compromise between brevity and precision while maintaining high standards of convergent validity. These results suggest that in its various versions, The DASS is reliable and adaptable for psychological assessment in clinical and research settings, confirming its usefulness for understanding the relationships between psychological distress and global well-being. In research contexts, the adoption of the DASS-9 would reduce the administration burden and likely increase participant involvement and reduce survey fatigue, without sacrificing the quality and accuracy of the assessments. Similarly, in clinical settings, where it is crucial to have rapid and effective measures to identify high levels of psychological distress, the DASS-9 is the ideal choice, due to both its brevity and proven convergence with measures of mental well-being. In summary, the DASS-9 offers the best compromise between brevity and comprehensiveness, making it a reliable and versatile instrument for assessing anxiety, stress, and depression. In contrast, the DASS-8, while offering sufficient indices of adjustment, only includes two items assessing stress, which may limit the ability to assess this construct adequately. The DASS-12, while offering more item coverage, is less parsimonious and does not offer significant advantages over the DASS-9.

### Theoretical Implications

From a theoretical standpoint, the present study’s findings reinforce the tripartite structure of negative affect in the DASS—stress, anxiety, and depression—even when the number of items is greatly reduced. The retention of clear subscale separability in all three brief forms underscores the robustness of the original DASS-21 factor structure and supports theories positing a common underlying vulnerability to negative emotionality alongside distinct symptom-specific processes. Moreover, the fact that the DASS-9 achieved optimal model fit suggests a “sweet spot” in scale design where removing redundancy maximizes construct validity without sacrificing content coverage. This may guide future scale development frameworks toward automated or theory-driven item-pruning techniques that preserve both breadth and depth of measurement.

### Practical Implications

From a practical standpoint, these abbreviated instruments offer several advantages for both research and clinical practice. In large-scale epidemiological surveys or experience-sampling studies—where participant burden must be minimized—the DASS-8 and DASS-9 can dramatically reduce administration time and survey fatigue while still providing reliable screening for psychological distress. In busy primary care or occupational health settings, the DASS-9 in particular could serve as a fast and economical triage tool to flag individuals at risk for clinically relevant levels of stress, anxiety, or depression, facilitating prompt referral for further assessment. Likewise, in digital mental-health platforms and smartphone apps where micro-assessment is key, embedding a short-item measure would likely enhance user engagement and compliance. Clinicians may also find the DASS-12 valuable when a slightly fuller symptom profile is desired—for example, to monitor subtle changes over the course of brief interventions—without reverting to longer versions of the DASS.

### Limitations and Future Directions

The findings of the present study must be interpreted while recognizing several limitations. First, the sample consisted of a self-selected convenience group from the broader Italian population, predominantly comprising females. This limits the generalizability of the findings to the entire Italian adult population, particularly to males and other underrepresented demographic groups. The gender imbalance may have influenced the results because previous research indicates that stress, anxiety, and depression can exhibit gender-related differences in patterns and severity. This potential bias could have affected understanding these psychological constructs from a gender perspective. Second, participants’ responses may have been influenced by social desirability bias, potentially distorting the accuracy of self-reported behaviors and attitudes. Third, the study’s cross-sectional design precluded an assessment of test-retest reliability, limiting insights into the temporal stability of the findings. Fourth, although the study examined divergent validity through its relationship with distinct constructs such as self-esteem, this aspect requires further investigation. Future studies could incorporate a broader range of theoretically distinct constructs, such as resilience and coping strategies, to ensure a more comprehensive assessment of divergent validity.

Fifth, while the study assessed convergent and divergent validity through its relationships with constructs such as perceived stress, life satisfaction, mental well-being, self-esteem, and negative affect, further research is needed to examine other constructs and variables. Future studies could include additional measures of related constructs, such as emotional dysregulation and psychological distress, to deepen the understanding of convergent and divergent validity.

Moreover, although the CFA models demonstrated acceptable fit and yielded clear, interpretable factor structures for the Italian DASS-8, DASS-9, and DASS-12, the study did not employ analytical techniques such as Exploratory Structural Equation Modeling (ESEM). ESEM’s ability to estimate cross-loadings can sometimes provide a more nuanced picture of inter-factor relations – especially when subscale correlations exceed .70. Future research should apply ESEM (and related bifactor-ESEM approaches) to these short forms in order to examine potential cross-loadings and further refine their latent structure in Italian samples.

Future research could also address these limitations by recruiting larger and more representative samples of Italian adults. Efforts should aim to achieve a more balanced gender distribution, as well as greater inclusion of other demographic subgroups. Stratified sampling techniques or targeted recruitment strategies could help ensure adequate representation of males and females and participants from diverse educational levels and age groups. Sixth, although the present study examined the psychometric properties of the short versions of the DASS-21 (i.e., 8-9,12 items), respecting the original validations (Ali et al., 2021; Kyriazos et al., 2018; Monteiro et al., 2023; Yusoff, 2013), future studies might consider testing other models, such as possible second-order solutions.

Employing nationally representative samples would enhance the generalizability of findings and provide a clearer picture of gender-related differences in stress, anxiety, and depression. Additionally, future studies should seek to validate the factorial solution proposed in the present study. Longitudinal designs involving diverse and large samples would allow for a deeper exploration of the relationships between variables examined in the present study. Investigating how stress, anxiety, and depression interact with other psychological constructs, such as social connectivity, could offer a more comprehensive understanding of their impact on overall well-being.

Finally, future research could explore potential differences in stress, anxiety, and depression across various age groups (e.g., adolescents and young adults) and educational levels. These analyses would help determine how demographic factors shape these psychological patterns, contributing to more targeted and effective interventions. In sum, addressing these areas would significantly enhance the robustness and applicability of future findings.

### Conclusions

The present study provides a comprehensive evaluation of the shortened versions of the DASS-21 (DASS-8, DASS-9, and DASS-12) among a sample of Italian adults, highlighting the importance of short and effective psychometric instruments for the assessment of negative emotional states such as stress, anxiety, and depression. The findings from both studies indicate that the DASS-9 model was optimal, exhibiting an optimal balance of indices of goodness of fit, and internal consistency. However, the DASS-8 and DASS-12 models may also be considered viable options because they both exhibited sufficient indices of goodness of fit. Moreover, all three short versions demonstrated strong validity and exhibited significant positive associations between stress, anxiety, depression, and negative affect, as well as significant negative correlations with mental well-being, positive affect, self-esteem, and life satisfaction. In sum, the study contributes to the extant literature by providing robust evidence for the efficacy of the shortened versions of DASS-21, which are suitable for applications requiring a rapid yet precise assessment of negative emotional states. Future studies could further investigate the generalizability of these findings to other populations and cultural contexts and explore the effectiveness of abbreviated versions of the DASS in clinical interventions and mental well-being monitoring programs.

## Supplemental Material

Supplemental Material - Psychometric Analyses of the Italian 8-Item, 9-Item, and 12-Item Versions of the Depression, Stress and Anxiety Scale Supplemental Material for Psychometric Analyses of the Italian 8-Item, 9-Item, and 12-Item Versions of the Depression, Stress and Anxiety Scale by Paolo Soraci, Mark D. Griffiths, Elena Del Fante, Renato Pisanti, Giulia Marafioti, Rocco Servidio, Elisa Chini, and Attila Szabo in Evaluation & the Health Professions

## Data Availability

The data are available upon reasonable request to the first author.

## Appendix

### Appendix A

Items from the DASS-21 to be used in the DASS-8, DASS-9, and DASS-12 (Bottesi et al., 2015):

**DASS 8:** *Depression*: DASS10, DASS13, DASS16 | Anxiety: DASS9, DASS15, DASS20 | *Stress*: DASS8, DASS12

**DASS 9:** *Depression*: DASS5, DASS10, DASS16 | Anxiety: DASS7, DASS9, DASS15 | *Stress*: DASS6, DASS11, DASS14

**DASS 12:** *Depression*: DASS10, DASS16, DASS17, DASS21 | Anxiety: DASS7, DASS9, DASS19, DASS20 | *Stress*: DASS1, DASS8, DASS11, DASS12
