# Supplementary material for: Psychometric Analyses of the Italian 8-Item, 9-Item, and 12-Item Versions of the Depression, Stress and Anxiety Scale
Source: Eval Health Prof. 2025 Sep 20;49(3):299–316. doi: 10.1177/01632787251380550 (PMC13379616; doi:10.1177/01632787251380550)
Supplement: Supplemental Material - Psychometric Analyses of the Italian 8-Item, 9-Item, and 12-Item Versions of the Depression, Stress and Anxiety Scale [file sj-pdf-1-ehp-10.1177_01632787251380550.pdf]

Supplementary Materials

Study 1

Table S1. Descriptive Statistics of DASS-21 items.

	Mean	SD	Skewness	SE	Kurtosis	SE
DASS1	1.389	0.887	0.201	0.106	-0.667	0.212
DASS2	1.004	0.955	0.555	0.106	-0.728	0.212
DASS3	1.151	0.970	0.392	0.106	-0.860	0.212
DASS4	0.964	1.005	0.644	0.106	-0.783	0.212
DASS5	1.347	0.995	0.179	0.106	-1.017	0.212
DASS6	1.430	0.993	0.061	0.106	-1.039	0.212
DASS7	0.750	0.988	1.026	0.106	-0.222	0.212
DASS8	1.623	0.975	-0.157	0.106	-0.961	0.212
DASS9	0.989	1.052	0.641	0.106	-0.891	0.212
DASS10	0.977	1.076	0.679	0.106	-0.900	0.212
DASS11	1.934	0.927	-0.411	0.106	-0.803	0.212
DASS12	1.689	0.979	-0.137	0.106	-1.022	0.212
DASS13	1.376	1.039	0.175	0.106	-1.133	0.212
DASS14	1.277	0.981	0.253	0.107	-0.952	0.213
DASS15	0.843	1.031	0.844	0.107	-0.640	0.213

Table S1. Descriptive Statistics of DASS-21 items.

	Mean	SD	Skewness	SE	Kurtosis	SE
DASS16	0.939	0.967	0.615	0.106	-0.772	0.212
DASS17	0.958	1.067	0.705	0.106	-0.852	0.212
DASS18	1.545	0.983	-0.010	0.106	-1.017	0.212
DASS19	1.181	1.062	0.382	0.106	-1.113	0.213
DASS20	0.902	0.998	0.747	0.106	-0.644	0.212
DASS21	0.760	1.005	1.032	0.106	-0.237	0.212

Note: SD= Standard deviation. SE= Standard Error.

Table S2. Factor loadings DASS-8

						95% Confidence Interval		
Factor	Item	Estimate	SE	z-value	p	Lower	Upper	Std. Est. (all)

Table S2. Factor loadings DASS-8

						95% Confidence Interval		
Factor	Item	Estimate	SE	z-value	p	Lower	Upper	Std. Est. (all)
Depression	DASS10	0.881	0.034	26.284	< .001	0.816	0.947	0.821
	DASS13	0.928	0.027	34.713	< .001	0.876	0.980	0.890
	DASS16	0.742	0.035	21.349	< .001	0.674	0.810	0.766
Anxiety	DASS9	0.844	0.037	22.873	< .001	0.772	0.917	0.803
	DASS15	0.836	0.037	22.433	< .001	0.763	0.909	0.811
	DASS20	0.767	0.036	21.553	< .001	0.698	0.837	0.769
Stress	DASS8	0.737	0.034	21.572	< .001	0.670	0.804	0.755
	DASS12	0.823	0.032	25.584	< .001	0.760	0.886	0.839

Note. SE= Standard Error. Estimate= Beta. Std. Est. (all)= Standardized Beta estimate

Table S3. Factor loadings DASS-9

						95% Confidence Interval		
Factor	Item	Estimate	SE	z-value	p	Lower	Upper	Std. Est. (all)
Depression	DASS5	0.713	0.040	17.639	< .001	0.634	0.792	0.716
	DASS10	0.867	0.042	20.860	< .001	0.785	0.948	0.809
	DASS16	0.769	0.038	20.336	< .001	0.695	0.843	0.795

Table S3. Factor loadings DASS-9

						95% Confidence Interval		
Factor	Item	Estimate	SE	z-value	p	Lower	Upper	Std. Est. (all)
Anxiety	DASS7	0.724	0.039	18.363	< .001	0.647	0.801	0.735
	DASS9	0.840	0.040	20.885	< .001	0.761	0.919	0.806
	DASS15	0.834	0.039	21.283	< .001	0.758	0.911	0.817
Stress	DASS6	0.722	0.040	18.118	< .001	0.643	0.800	0.728
	DASS11	0.684	0.037	18.345	< .001	0.611	0.757	0.735
	DASS14	0.744	0.039	19.049	< .001	0.667	0.821	0.756

Note. SE= Standard Error. Estimate= Beta. Std. Est. (all)= Standardized Beta estimate

Table S4. Factor loadings DASS-12

						95% Confidence Interval		
Factor	Item	Estimate	SE	z-value	p	Lower	Upper	Std. Est. (all)
Depression	DASS10	0.919	0.032	28.370	< .001	0.856	0.983	0.854
	DASS16	0.761	0.035	21.516	< .001	0.692	0.830	0.788
	DASS17	0.908	0.034	26.793	< .001	0.841	0.974	0.848
	DASS21	0.818	0.038	21.808	< .001	0.745	0.892	0.817
Anxiety	DASS7	0.690	0.039	17.484	< .001	0.612	0.767	0.699
	DASS9	0.825	0.037	22.035	< .001	0.752	0.899	0.784
	DASS19	0.791	0.035	22.706	< .001	0.723	0.859	0.746
	DASS20	0.754	0.037	20.489	< .001	0.681	0.826	0.761

Table S4. Factor loadings DASS-12

Factor	Item	Estimate	SE	z-value	p	95% Confidence Interval Lower	Upper	Std. Est. (all)
Stress	DASS1	0.646	0.033	19.853	< .001	0.583	0.710	0.731
	DASS8	0.763	0.032	24.024	< .001	0.701	0.826	0.782
	DASS11	0.790	0.027	28.760	< .001	0.736	0.844	0.850
	DASS12	0.827	0.030	27.933	< .001	0.769	0.885	0.846

Note. SE= Standard Error. Estimate= Beta. Std. Est. (all)= Standardized Beta estimate

Table S5. Factor loadings DASS-21

Factor	Item	Estimate	SE	z-value	p	95% Confidence Interval Lower	Upper	Std. Est. (all)
Depression	DASS3	0.703	0.037	19.006	< .001	0.631	0.776	0.726
	DASS5	0.684	0.039	17.569	< .001	0.608	0.760	0.687
	DASS10	0.898	0.038	23.326	< .001	0.822	0.973	0.836
	DASS13	0.894	0.036	24.498	< .001	0.822	0.965	0.861
	DASS16	0.757	0.036	21.179	< .001	0.686	0.827	0.784
	DASS17	0.884	0.038	23.018	< .001	0.809	0.959	0.829
	DASS21	0.781	0.037	20.935	< .001	0.708	0.855	0.778
Stress	DASS1	0.660	0.034	19.638	< .001	0.594	0.726	0.744
	DASS6	0.722	0.038	19.018	< .001	0.648	0.796	0.728
	DASS8	0.776	0.036	21.704	< .001	0.706	0.846	0.797
	DASS11	0.760	0.034	22.642	< .001	0.694	0.826	0.820

Table S5. Factor loadings DASS-21

Factor	Item	Estimate	SE	z-value	p	95% Confidence Interval Lower	Upper	Std. Est. (all)
Anxiety	DASS12	0.805	0.035	22.672	< .001	0.735	0.874	0.822
	DASS14	0.724	0.037	19.332	< .001	0.651	0.797	0.739
	DASS18	0.775	0.036	21.276	< .001	0.703	0.846	0.788
	DASS2	0.459	0.040	11.386	< .001	0.380	0.538	0.482
	DASS4	0.814	0.037	22.164	< .001	0.742	0.886	0.811
	DASS7	0.692	0.038	18.045	< .001	0.617	0.768	0.702
	DASS9	0.803	0.040	20.293	< .001	0.725	0.880	0.764
	DASS15	0.889	0.036	24.448	< .001	0.818	0.961	0.865
	DASS19	0.850	0.039	21.706	< .001	0.773	0.927	0.801
	DASS20	0.729	0.038	19.085	< .001	0.654	0.804	0.731

Note. SE= Standard Error. Estimate= Beta. Std. Est. (all)= Standardized Beta estimate

Study 2

Table S6. Descriptive Statistics of DASS-21 items.

	Mean	SD	Skewness	SE	Kurtosis	SE
DASS1	2.402	1.281	0.462	0.136	-0.949	0.271
DASS2	1.956	1.150	0.854	0.136	-0.432	0.271
DASS3	1.866	1.134	1.146	0.136	0.275	0.271
DASS4	1.863	1.199	1.153	0.136	0.086	0.271
DASS5	2.530	1.405	0.415	0.136	-1.140	0.271
DASS6	2.520	1.374	0.321	0.136	-1.239	0.271
DASS7	1.776	1.227	1.405	0.136	0.680	0.271
DASS8	2.748	1.419	0.143	0.136	-1.345	0.271
DASS9	2.162	1.394	0.802	0.136	-0.800	0.271
DASS10	2.109	1.362	0.900	0.136	-0.549	0.271
DASS11	3.377	1.483	-0.459	0.136	-1.228	0.271
DASS12	3.037	1.429	-0.124	0.136	-1.357	0.271
DASS13	2.701	1.478	0.234	0.136	-1.403	0.271
DASS14	2.374	1.288	0.546	0.136	-0.864	0.271
DASS15	2.308	1.477	0.621	0.136	-1.129	0.271

Table S6. Descriptive Statistics of DASS-21 items.

	Mean	SD	Skewness	SE	Kurtosis	SE
DASS16	2.121	1.311	0.828	0.136	-0.597	0.271
DASS17	2.262	1.432	0.670	0.136	-1.043	0.271
DASS18	2.885	1.399	0.083	0.136	-1.283	0.271
DASS19	2.436	1.395	0.424	0.136	-1.218	0.271
DASS20	2.131	1.333	0.810	0.136	-0.724	0.271
DASS21	2.003	1.395	1.071	0.136	-0.327	0.271

Note: SD= Standard deviation. SE= Standard Error.

Table S7. Factor loadings DASS-8

						95% Confidence Interval		
Factor	Indicator	Estimate	Std. Error	z-value	p	Lower	Upper	Std. Est. (all)
Depression	DASS10	1.091	0.064	16.933	< .001	0.965	1.217	0.803
	DASS13	1.369	0.064	21.307	< .001	1.243	1.495	0.928
	DASS16	0.977	0.064	15.265	< .001	0.851	1.102	0.746
Anxiety	DASS9	1.083	0.068	15.986	< .001	0.950	1.216	0.778
	DASS15	1.160	0.071	16.237	< .001	1.020	1.300	0.786
	DASS20	1.129	0.062	18.201	< .001	1.008	1.251	0.849
Stress	DASS8	1.113	0.069	16.186	< .001	0.978	1.248	0.785
	DASS12	1.290	0.065	19.705	< .001	1.162	1.419	0.904

Table S8. Factor loadings DASS-9

						95% Confidence Interval		

Factor	Indicator	Estimate	Std. Error	z-value	p	Lower	Upper	Std. Est. (all)
Depression	DASS5	1.027	0.071	14.487	< .001	0.888	1.165	0.732
	DASS10	1.097	0.067	16.475	< .001	0.967	1.228	0.807
	DASS16	0.974	0.066	14.761	< .001	0.845	1.103	0.744
Anxiety	DASS7	0.811	0.064	12.691	< .001	0.686	0.937	0.662
	DASS9	1.124	0.068	16.645	< .001	0.992	1.257	0.808
	DASS15	1.171	0.072	16.269	< .001	1.030	1.312	0.794
Stress	DASS6	1.125	0.065	17.431	< .001	0.999	1.252	0.820
	DASS11	1.171	0.071	16.523	< .001	1.032	1.310	0.791
	DASS14	1.089	0.060	18.305	< .001	0.973	1.206	0.847

Table S9. Factor loadings DASS-12

95% Confidence Interval

						95% Confidence Interval		
Factor	Indicator	Estimate	Std. Error	z-value	p	Lower	Upper	Std. Est. (all)
Depression	DASS10	1.181	0.062	19.141	< .001	1.060	1.302	0.869
	DASS16	1.026	0.063	16.295	< .001	0.902	1.149	0.783
	DASS17	1.168	0.067	17.383	< .001	1.037	1.300	0.817
	DASS21	1.161	0.065	17.910	< .001	1.034	1.289	0.834
Anxiety	DASS7	0.768	0.064	11.979	< .001	0.642	0.894	0.627
	DASS9	1.081	0.067	16.020	< .001	0.949	1.213	0.777
	DASS19	1.036	0.069	15.052	< .001	0.901	1.171	0.743
	DASS20	1.128	0.062	18.201	< .001	1.006	1.249	0.847
Stress	DASS1	1.023	0.060	16.921	< .001	0.905	1.142	0.800
	DASS8	1.146	0.067	17.199	< .001	1.016	1.277	0.809
	DASS11	1.300	0.066	19.627	< .001	1.170	1.430	0.878
	DASS12	1.297	0.062	20.774	< .001	1.175	1.420	0.909

Table S10. Factor loadings DASS-21

Factor	Indicator	Estimate	Std. Error	z-value	p	Lower	Upper	Std. Est. (all)
Depression	DASS3	0.749	0.057	13.098	< .001	0.637	0.861	0.662
	DASS5	0.977	0.070	14.001	< .001	0.840	1.113	0.696
	DASS10	1.142	0.062	18.355	< .001	1.020	1.264	0.840
	DASS13	1.318	0.065	20.282	< .001	1.191	1.446	0.893
	DASS16	1.029	0.062	16.584	< .001	0.907	1.150	0.786
	DASS17	1.140	0.067	16.953	< .001	1.008	1.271	0.797
	DASS21	1.113	0.066	16.979	< .001	0.985	1.242	0.799
Anxiety	DASS2	0.746	0.059	12.684	< .001	0.630	0.861	0.649
	DASS4	0.856	0.059	14.402	< .001	0.739	0.972	0.715
	DASS7	0.813	0.062	13.050	< .001	0.691	0.935	0.663
	DASS9	1.105	0.066	16.754	< .001	0.975	1.234	0.794
	DASS15	1.164	0.070	16.613	< .001	1.027	1.301	0.789
	DASS19	1.049	0.068	15.518	< .001	0.916	1.181	0.753
	DASS20	1.086	0.062	17.459	< .001	0.964	1.208	0.816
Stress	DASS1	1.021	0.060	17.073	< .001	0.904	1.139	0.799
	DASS6	1.124	0.063	17.724	< .001	0.999	1.248	0.819
	DASS8	1.168	0.065	17.917	< .001	1.041	1.296	0.825
	DASS11	1.275	0.066	19.183	< .001	1.145	1.405	0.861
	DASS12	1.249	0.063	19.686	< .001	1.124	1.373	0.875

Table S10. Factor loadings DASS-21

						95% Confidence Interval		
Factor	Indicator	Estimate	Std. Error	z-value	p	Lower	Upper	Std. Est. (all)
	DASS14	1.044	0.060	17.481	< .001	0.927	1.161	0.811
	DASS18	1.191	0.063	18.891	< .001	1.068	1.315	0.853

Table S11. Main socio-demographic characteristics of the participants of the two studies.

**Characteristic**	**Study 1 (N = 541)**	**Study 2 (N = 323)**
**Mean age (SD)**	35.36 (±12.14)	34.38 (±12.83)

**Age range**	19 - 71 years	18 - 80 years
**Gender**	77.0% female (n=416)	75.0% female (n=242)
	23.0% male (n=124)	25.0% male (n=80)
**Marital status**	37.5% married/cohabiting (n=203)	26.31% married/cohabiting (n=85)
	28.3% engaged (n=153)	Not reported
	25.7% single (n=139)	65.01% single (n=210)
	9.5% other (n=51)	8.64% other (n=28)
**Education level**	54.7% high school (n=296)	46.13% high school (n=149)
	43.3% university degree or higher (n=234)	52.93% university degree or higher (n=171)
	2% lower secondary diploma (n=10)	3.0% lower secondary diploma (n=3)

Study 1 and 2 HTMT (Heterotrait-Monotrait ratio)

Table S12. Study 1 - Heterotrait-monotrait ratio DASS-8

Depression	Anxiety	Stress
1.000		
0.797	1.000	
0.831	0.834	1.000

Table S14. Study 1 - Heterotrait-monotrait ratio DASS-12

Depression	Anxiety	Stress
1.000		
0.758	1.000	
0.745	0.839	1.000

Table S15. Study 1 - Heterotrait-monotrait ratio DASS-21

Depression	Stress	Anxiety
1.000		
0.850	1.000	
0.808	0.848	1.000

Table S16. Study 2 - Heterotrait-monotrait ratio DASS-21

Depression	Stress	Anxiety
1.000		
0.871	1.000	
0.883	0.895	1.000

Table S17. Study 2 - Heterotrait-monotrait ratio DASS-8

Depression	Stress	Anxiety
1.000		
0.806	1.000	
0.850	0.839	1.000

Table S18. Study 2 - Heterotrait-monotrait ratio DASS-9

Depression	Anxiety	Stress
1.000		
0.849	1.000	
0.848	0.837	1.000

Table S19. Study 2 - Heterotrait-monotrait ratio DASS-12

Depression	Anxiety	Stress
1.000		
0.849	1.000	
0.768	0.839	1.000

Study 1 and Study 2

Image S1. DASS-8 model plot

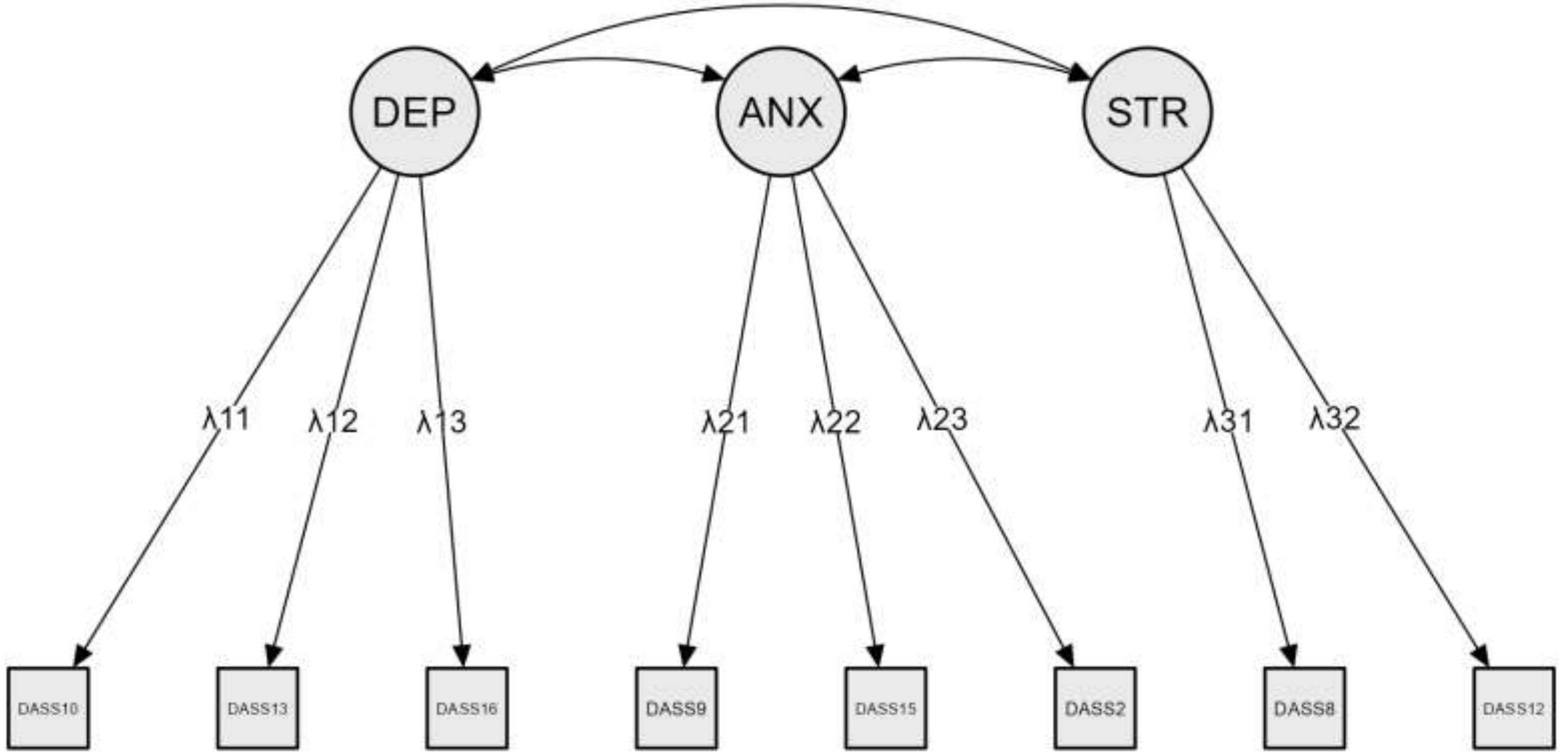

Note: DEP= Depression, ANX= Anxiety, STR= Stress.

Image S2. DASS-9 model plot

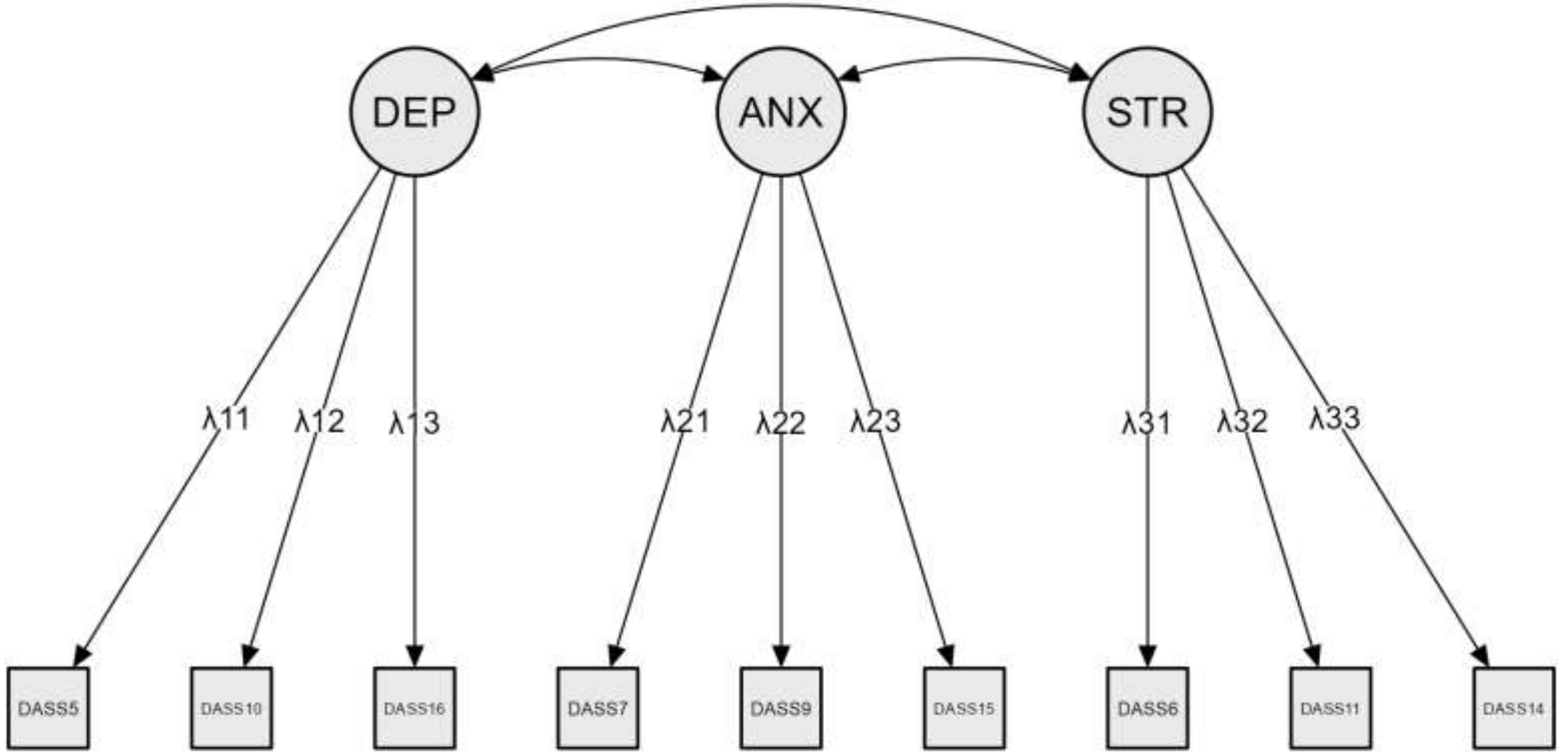

Note: DEP= Depression, ANX= Anxiety, STR= Stress.

Image S3. DASS-12 model plot

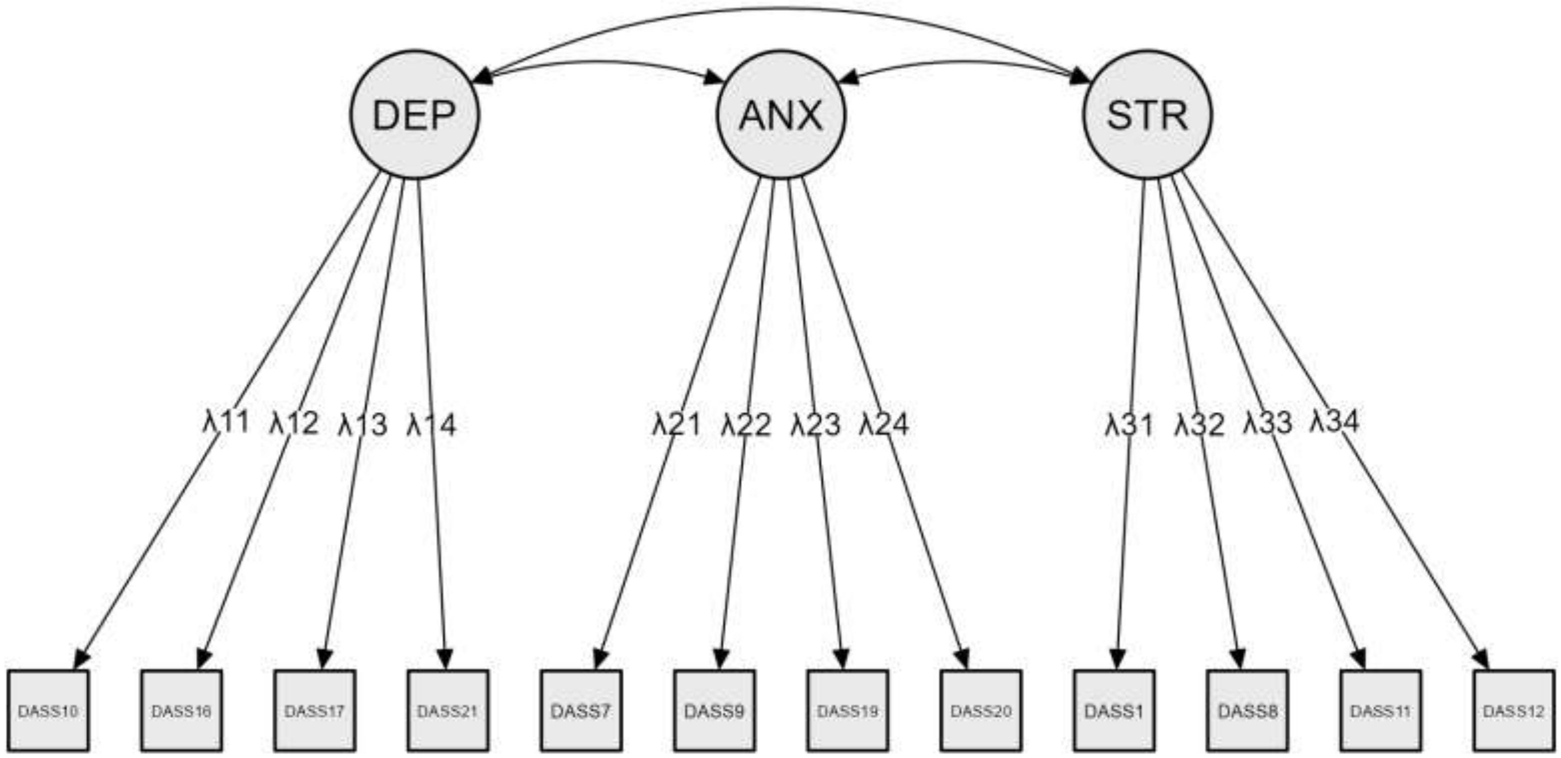

Note: DEP= Depression, ANX= Anxiety, STR= Stress.
